# S100A proteins in the pathogenesis of experimental corneal neovascularization

**Published:** 2010-10-31

**Authors:** Changyou Li, Feng Zhang, Yiqiang Wang

**Affiliations:** 1 Qingdao University-SEI Joint Ophthalmology Program, Qingdao, China; 2 Shandong Provincial Key Lab of Ophthalmology, Shandong Eye Institute, Qingdao, China

## Abstract

**Purpose:**

The S100A protein family is involved in various inflammatory processes. Two of its members, S100A4 and A13, are thought to be pro-angiogenic in tumor development. This study examines whether S100A proteins are involved in the pathogenesis of inflammation-associated corneal neovascularization (CorNV).

**Methods:**

We used 10–0 nylon suture- (S) or chemical burn (CB) -induced CorNV models for a microarray analysis of the genome-wide expression pattern. At different time points after suturing, we conducted histopathological examinations to detect the infiltration of inflammatory cells into the corneal stroma. Representative members of the S100A family (S100A4, S100A6, S100A8, S100A9, and S100A13), pro-inflammatory cytokines (IL-1β, IL-6, transforming growth factor β1, and MIP-2), and pro-angiogenic factors (fibroblast growth factor and vascular endothelial growth factor) were detected with reverse-transcription quantitative PCR (RT-QPCR). We used immunofluorescence to monitor neutrophil or macrophage infiltration and S100A8 or S100A9 protein deposition in neovascularized corneas. Antibody-mediated neutrophil depletion or S100A8 depletion in mice was performed to evaluate the role of neutrophils and S100A proteins in suture-induced corneal neovascularization (S-CorNV).

**Results:**

Microarray profiling revealed that S100A4, S100A6, S100A8, S100A9, and S100A13 were upregulated in both CorNV models, with S100A8 and S100A9 manifesting the most significant changes compared to the normal animals. An RT-QPCR assay of these *S100A* genes and cytokine genes in the S-CorNV corneas showed that the changes were time-dependent, reaching the apex at day 5. Immunofluorescence analysis demonstrated that neutrophils and macrophages produce S100A8 and S100A9. The depletion of neutrophils beginning one day before S-CorNV induction decreased disease severity and S100A8/S100A9 deposition in the neovascularized corneas. The extent of upregulation of other detected S100A genes and pro-inflammatory or pro-angiogenic genes was also decreased by neutrophil depletion. Subconjunctival administration of S100A8 antibodies also significantly inhibited the growth of vessels and inflammation in the S-CorNV model.

**Conclusions:**

We determined that S100A proteins are involved in the inflammatory CorNV model and that S100A8 or S100A9 in particular might be employed as targets in managing diseases involving this pathological process.

## Introduction

Corneal transparency is necessary for normal vision and may be compromised by pathological factors such as infection, trauma, degeneration, corneal graft rejection, contact lens-related hypoxia, neurotrophic ulceration, aniridia, and limbal stem cell deficiency [1]. One of the main complications of such conditions is neovascularization, here referring to the growth of vessels in the originally avascular area of the cornea [2]. The process and mechanism of corneal neovascularization (CorNV) can be complicated. Depending on different pathogenic initiators, inflammation is often present in neovascularized corneas and is thus called inflammation-associated (or -induced) neovascularization. In that context, macrophages, myelomonocytes, and neutrophils are the most commonly seen cellular populations, all of which are main sources of pro-angiogenic or anti-angiogenic factors [3-5]. They also produce other cytokines, chemokines, or enzymes that modulate the functions of cells involved in angiogenesis [6-9].

The action of vascular endothelium growth factor (VEGF) on vascular endothelial cells has been characterized as the last and most common step in various pro-angiogenic pathways. Various strategies targeting vascular endothelium growth factor A (VEGFA) have been extensively tested in neovascularization-related diseases. The search for other molecules that could be used as targets to manage CorNV is ongoing. One strategy focuses on the inflammation process that occurs before neovascularization. In searching for potential targets in the case of inflammation-induced CorNV, a group of proteins—the S100 family—attracted our attention. Representing one of the largest subfamilies of the EF-hand calcium-binding proteins with at least 19 different members [10], S100 proteins interact with other proteins to modulate a variety of biologic functions and are thus related to various diseases, many of which involve inflammation, innate immunity, tissue damage, and wound healing [11,12]. Apart from the possible crosstalk between S100 proteins and pro- or anti-angiogenic factors, S100A4 and A13 have been reported to participate directly in the angiogenic process in other tissues, such as cancer tissues [13-17]. In the present study, we first used microarray analysis to profile the genes that were differentially regulated in experimental CorNV models. We then focused on the possible role of S100A proteins in the pathogenesis of CorNV.

## Methods

### Corneal neovascularization models

Six- to eight-week-old Balb/c mice were purchased from Beijing Pharmacology Institute, Chinese Academy of Medical Sciences (Beijing, China). We complied with the Association for Research in Vision and Ophthalmology (ARVO) Statement for the Use of Animals in Ophthalmic and Vision Research throughout the study. The procedures were performed under anesthesia with intraperitoneal chlorpromazine (Harvest Pharmaceutical, Shanghai, China) and ketamine (Heng Rui Medicine, Jiangsu, China). There was also a topical application of 0.5% proparacaine hydrochloride (Alcon-Couvreur, Puurs, Belgium) for topical anesthesia. For the suture-induced CorNV (S-CorNV), three interrupted 10–0 polypropylene sutures (MANI Inc., Togichi, Japan) were placed following the protocol of other researchers [18], with some modification. In detail, a 2 mm trephine was pressed slightly against the central cornea to produce a circular mark. Three stitches were evenly placed, with both points of each stitch going in and out of the cornea along the mark. The sutures went through the epithelial and stromal layers, but did not penetrate the endothelial layer. For chemical burn-induced CorNV (CB-CorNV), a piece of disk-shaped filter paper 2.0 mm in diameter was immersed in 1 mol/l NaOH solution for 15 s and placed on the central corneal surface for 50 s to produce a circular burn, followed by immediate washing with 30 ml of 0.9% saline. Only the right eye of each mouse was used for CorNV induction. Photographs of the corneas were taken with a camera mounted on a slit-lamp microscope and the neovascularization score was calculated as previously described [18]. Briefly, CorNV was graded between 0 and 3, with increments of 0.5, using a grid system for each quadrant based on the centripetal extent of the neovascular branches from the limbus. Scores for each quadrant were summed to obtain the CorNV score (range 0 to 12) for each eye. At predetermined time points, the corneas were harvested for microarray analysis, reverse transcription-quantitative PCR (RT-QPCR), histology, or immunofluorescence assays, as described below.

### Isolation of total RNA

For the extraction of total RNA used for either microarray analysis or RT-QPCR, the corneas were excised using a 2 mm diameter trepan and placed in ice-cold TRIzol reagent (Invitrogen, Gaithersburg, MD) at desired time points after CorNV induction. Five model corneas from each group of mice were pooled and the untreated corneas from the same mice were used as controls. Total RNA was extracted using isopropanol precipitation and was purified using NucleoSpin^®^ RNA clean-up columns (MACHEREY-NAGEL, Düren, Germany). The quality and integrity of the RNA was confirmed by denaturing aldehyde agarose electrophoresis.

### Microarray analysis

Dual cRNA labeling with Cy5 and Cy3 fluorescence and microarray hybridizations were performed by Capital Bio Corporation using Capital Bio cRNA labeling kits and the Capital Bio 36 K Mouse Genome Oligo Array (Capital Bio, Beijing, China) [19]. In brief, the array comprises 35,852 70-mer oligonucleotide probes representing approximately 25,000 genes of Mouse Genome Version 4.0 (Operon Biotechnologies, Huntsville, AL). The Cy5 and Cy3 were used to label cRNA of the experimental and control groups. Two or three replicate arrays were used for each time point for each model. After hybridization, the arrays were scanned using a LuxScan 10KA (Capital Bio) and signals were processed using LuxScan 3.0 software (Capital Bio). Intra-array normalization was done using the LOcally WEighted Scatterplot Smoothing (LOWESS) normalization method and inter-array normalization of the whole data set was performed according to the global means of Cy5 and Cy3 signals [20]. Normalized signal intensities were compared between the experimental and control samples, and the ratios were used to reflect the change in the expression level of each gene.

### RT-QPCR analysis

One microgram total RNA from each sample was reverse transcribed into cDNA using a PrimeScript RT Reagent Kit (Takara, Shiga, Japan), following the manufacturer’s instructions. Quantitative PCR was performed with cDNA corresponding to 62.5 ng RNA using the Taqman method with proper primers and probes (Table 1). Apart from the S100A proteins, representative pro-inflammatory cytokines (IL-1β, IL-6, macrophage-inflammatory protein-2 (MIP-2), and transforming growth factor β1 (TGF)), and pro-angiogenic factors (VEGFA and fibroblast growth factor (FGF)) were also studied. The reactions were run in an ABI 7500 Detection System (Applied Biosystems, Foster City, CA) for 10 min at 95 °C followed by 40 cycles of amplification for 15 s at 95 °C and 1 min at 60 °C. The raw data was analyzed using SDS 7500 software (Applied Biosystems) and Ct values for each gene in each sample were obtained for further analysis. The RPL5 gene was used as the reference gene for quantification in this assay. Each sample was run in duplicate. The relative level of the gene of interest was obtained using the Equation 1/2^(Ct for gene - Ct for RPL5)^, and the final relative expression ratio of each gene in each group was obtained by the geometric mean of five samples in each group.

**Table 1:** Sequences of primers and probes used for RT-qPCR assay.

**Gene symbol (accession number)**	**Primer sequence (5′-3′)**	**Amplicon (bp)**
*S100A4* (NM_011311)	F- GGACAGCAACAGGGACAATGA	101 bp
	R- TATCTGGGCAGCCCTCAAAG	
	P- AGTACTGTGTCTTCCTGTCCTGCATTGCCA	
*S100A6* (NM_011313)	F- GTACTCTGGCAAGGAAGGTGACA	101 bp
	R- CAGCATCCTGCAGCTTGGA	
	P- AAGGAGCTGAAGGAGTTGATCCAGAAG	
*S100A8* (NM_013650)	F- CGAAAACTTGTTCAGAGAATTGGA	81 bp
	R- ACTTTTATCACCATCGCAAGGAA	
	P- ATCAATAGTGACAATGCAATTAACTTCGA	
*S100A9* (NM_009114)	F- GAGCGCAGCATAACCACCAT	101 bp
	R- TCCACCATTTGTCTGAATTCCTT	
	P- ATCGACACCTTCCATCAATACTCTAGGA	
*S100A13* (NM_009113)	F- CTCAAGGACGTGGGCTCTCT	101 bp
	R- AGCTCTCCAATCAGTCTCCAGTATTC	
	P- ATGAAAAGATGAAGACCTTGGATGTGA	
*IL-1β* (NM_008361)	F- AGATGAAGGGCTGCTTCCAA	81 bp
	R- TGATGTGCTGCTGCGAGATT	
	P- TGACCTGGGCTGTCCTGATGA	
*IL-6* (NM_031168)	F- GTTGCCTTCTTGGGACTGATG	91 bp
	R- TGGGAGTGGTATCCTCTGTGAA	
	P- TGACAACCACGGCCTTCCCTACTTCA	
*MIP-2* (NM_009140)	F- GAACATCCAGAGCTTGAGTGTGA	86 bp
	R- CTTTTTGACCGCCCTTGAGA	
	P- CCCCAGGACCCCACTGCGC	
*VEGFA* (NM_009505)	F- GCTACTGCCGTCCGATTGAG	86 bp
	R- CACACAGGACGGCTTGAAGA	
	P- CCTGGTGGACATCTTCCAGGAGTACCC	
*FGF2* (NM_008006)	F- AGAGCGACCCACACGTCAA	86 bp
	R- AAGGTACCGGTTGGCACACA	
	P- TCCAAGCAGAAGAGAGAGGAGTTGTGT	
*TGFβ1* (NM_011577)	F- ACGGAATACAGGGCTTTCGA	86 bp
	R- GCTGATCCCGTTGATTTCCA	
	P- TCAGCGCTCACTGCTCTTGTG	
*RPL5* (NM_016980)	F- GGAAGCACATCATGGGTCAGA	70 bp
	R- TACGCATCTTCATCTTCCTCCATT	
	P- TGTGGCAGACTACATGCGCTACC	

In the “Primer” column, “F” indicates forward primer, “R” indicates reverse primer, and “P” indicates probe sequence.

### Histological and immunohistochemical analysis

Corneas removed from various groups of mice were subjected to fixation in 10% paraformaldehyde followed by regular Hematoxylin-Eosin (HE) staining, or to cryosection and immunostaining. In brief, paraffin-embedded corneal tissues were continually cut into 4 μm thick slices and stained with HE. Cryopreserved corneas in Optimum Cutting Temperature formulation (Sakura Finetek, Tokyo, Japan) were sectioned and labeled with goat anti-mouse S100A8 or goat anti-mouse S100A9 in combination with rabbit anti-mouse Gr-1 or rabbit anti-mouse F4/80 at 4 °C overnight. After three washes with PBS-Tween buffer, a mixture of PE conjugated bovine anti-goat IgG and FITC conjugated bovine anti-rabbit IgG was applied for 30 min. All antibodies were products of Santa Cruz Biotechnology Inc. (Santa Cruz, CA) and the concentrations of antibodies were chosen based on the manufacturer’s instructions. The sections were observed using an E800 fluorescence microscope (Nikon, Tokyo, Japan) with proper filters.

### Neutrophil depletion study

To deplete neutrophils in vivo and study the effect on CorNV pathogenesis, purified anti-mouse Gr-1 antibody (RB6–8C5; eBioscience, San Diego, CA) was injected intraperitoneally (0.5 mg in 0.2 ml per mouse) [21] every other day starting one day before S-CorNV induction. Control animals received an equivalent volume of PBS. Suture CorNV induction and molecular studies of the neutrophil-depleted mice were performed as described above to study the potential effect of neutrophil depletion on the development of S-CorNV.

### Subconjunctival injection of anti-S100A8 mAb

To block the activity of S100A8 during CorNV development, 20 μg of anti-S100A8 monoclonal antibody (clone 8H150; LifeSpan BioSciences, Seattle, WA) in 5 μl of PBS was injected into the subconjunctival space three times—immediately after suture placement and then on days 2 and 4 after suture placement. Equivalent PBS was injected into the control eyes. The eyes were monitored every other day and enucleated on day 7 for histological analysis.

### Statistical analysis

Statistical analysis was performed where applicable using Student’s two-tailed *t* tests. P values >0.05 indicated a significant difference between the groups.

## Results and Discussion

### Upregulation of *S100A* gene expression in corneas upon CorNV induction

In our pilot studies, we found that S-CorNV developed rapidly around day 5 and peaked around day 10, while CB-induced CorNV reached similar levels at days 6 and 14, respectively. By comparing the gene expression profiles of both models at these stages, we found that some members of the S100A protein family showed significant changes compared to the normal animals. The changes for all genes as a set was comparable in the two models (Table 2). In particular, *S100A8*, also known as myloid-related protein 8 (*MRP8*), and *S100A9* (*MRP14*) increased abundantly at both time points in both CorNV models, as detected by the microarray assay. The *S100A4* was also upregulated, but to a lesser extent, while *S100A3* and *S100A13* only slighted increased. The remaining *S100A* members did not show significant changes. The RT-QPCR assay of *S100A* gene expression in the S-CorNV model revealed that the changes of all *S100A* genes manifested in a similar time-dependent manner, peaking around day 5 for all genes (Table 3). It is noteworthy that, as reported in other situations [22,23], the fold changes of regulated genes obtained using RT-QPCR were much higher than those obtained using the microarray. While both *S100A4* [13-15] and *S100A13* [16,17] are thought to participate in the angiogenic process in other tissues, neither *S100A8* or *S100A9* have been found to be involved in such processes in any situation. We therefore focused our attention on *S100A8* and *S100A9*.

**Table 2:** Expression level changes of S100A and inflammatory or pro-angiogenic genes detected by microarray.

**Gene**	**S-CorNV-Day 5**	**S-CorNV-Day 10**	**CB-CoNV-Day 6**	**CB-CorNV-Day 14**
*S100A1*	1.72±1.25^a^	2.00±1.11	0.90±0.45	1.41±1.20
*S100A3*	1.63±1.21	1.47±1.06	2.15±1.15	3.10±1.01
*S100A4*	5.13±1.17	3.44±1.15	3.41±1.35	3.09±1.16
*S100A6*	0.79±1.12	1.25±1.13	0.64±1.12	0.74±1.01
*S100A8*	11.50±1.12	7.76±1.29	9.08±1.07	7.31±1.43
*S100A9*	45.35±1.36	19.55±1.35	15.97±1.19	18.51±1.93
*S100A10*	1.20±1.12	1.26±1.70	1.07±1.06	1.22^b^
*S100A11*	1.02±1.17	1.04±1.20	1.51±1.00	1.45±1.14
*S100A13*	2.77±1.10	2.13±1.05	1.56±1.12	1.73^b^
*S100A14*	1.15±1.26	1.00±1.22	0.88±1.18	0.81±1.02
*S100A15*	0.96±1.31	0.72±1.16	0.44±1.02	0.45±1.14
*S100A16*	1.06±1.18	1.01±1.03	0.74±1.04	0.82±1.01
*IL1β*	22.61±1.45	12.37±1.35	9.87±1.20	11.46±1.06
*IL6*	259.50±5.99	NA^c^	NA	NA
*MIP2*	35.48±1.68	NA	9.30±1.48	NA
*VEGFA*	3.63±1.63	2.22±1.81	2.01±1.95	1.40±1.43
*FGF2*	NA	NA	NA	NA
*TGFβ1*	0.62±1.41	0.83±1.34	1.53±1.55	0.56±1.47

^a^There were three replicate arrays for S-CorNV-Day 5 and CB-CorNV-Day 6 groups, and two replicate arrays for S-CorNV-Day 10 and CB-CorNV-Day 14 groups. Shown are geometric means (±standard deviations) of fold changes of gene expression in each group. ^b^Only one array produced a valid fold change for this gene in this group. To get a valid fold change for each array, both the experimental sample and the control sample were required to give valid signals after normalization of the raw signals. Failing to obtain an effective signal for a specific gene in any sample lead to the absence of a fold change in that array. ^c^NA, not available. In the dual fluorescence microarray format, an actual ratio will not be available when the signal for either the control or experimental sample is classified as undetectable, even if the signal for its component was apparently much higher or lower. Without exception, all NAs in this table were derived because control samples in all arrays in a group gave values below the threshold of detection after standardization.

**Table 3:** Relative quantification of gene expression in S-CorNV model detected by RT-QPCR.

**Gene**	**Day 1**	**Day 3**	**Day 5**	**Day 7**	**Day 10**	**Day 14**	**Day 20**
*S100A4*	1.60±0.12	2.73±0.09	3.78±0.19	4.04±0.22	2.30±0.20	2.43±0.08	1.14±0.03
*S100A6*	1.01±0.05	1.00±0.04	0.82±0.05	1.05±0.05	1.17±0.28	1.61±0.06	1.34±0.08
*S100A8*	79.45±2.21	617.01±151.71	1892.05±54.47	626.98±54.69	190.13±7.99	57.94±4.19	6.89±0.18
*S100A9*	156.02±18.05	628.19±18.07	1604.94±82.88	1089.40±28.17	432.86±23.56	102.07±3.88	24.85±1.51
*S100A13*	1.06±0.10	2.09±0.14	2.40±0.30	2.00±0.04	1.50±0.17	1.69±0.14	1.24±0.05
*IL-1β*	27.92±1.63	36.49±2.35	98.57±4.50	58.13±0.82	46.92±5.02	45.10±2.52	3.90±0.29
*IL-6*	12.01±1.74	23.24±2.74	72.38±7.15	63.81±3.95	19.35±1.34	17.50±1.67	5.14±0.67
*MIP-2*	14.88±0.41	13.91±0.59	34.46±2.60	26.73±1.83	16.38±0.71	11.46±1.25	1.07±0.12
*VEGFA*	13.84±1.76	14.37±0.24	21.21±2.32	15.43±0.59	9.51±1.56	6.18±0.33	4.24±0.24
*FGF2*	6.26±0.21	9.33±1.46	9.61±1.39	5.31±0.32	4.00±0.49	2.24±0.57	1.82±0.25
*TGFβ1*	0.86±0.06	1.38±0.09	2.97±0.31	1.96±0.12	1.70±0.03	1.51±0.20	1.56±0.07

We also measured two classical pro-angiogenic factors (*VEGFA* and *FGF*) and several factors known to be produced by macrophages or neutrophils, including *IL-1β*, *IL-6*, *MIP-2*, and *TGFβ1*. Microarrays and RT-QPCR have revealed that most other factors manifested change patterns similar to that of *S100A8* or *S100A9* in terms of the time course, namely reaching maximum upregulation at day 5 after S-CorNV induction (Table 2 and Table 3). Specifically, *S100A8* and *S100A9* gave much higher upregulation folds than all other *S100A* genes, inflammatory genes, or pro-angiogenic genes. Thus, the significance of the *S100A8* and *S100A9* expression changes, as well as the interactions between *S100A8* or *S100A9* and those traditional angiogenic factors, deserve further study.

### Accumulation of S100A8 and S100A9 proteins in neovascularized corneas

**Figure 1:**
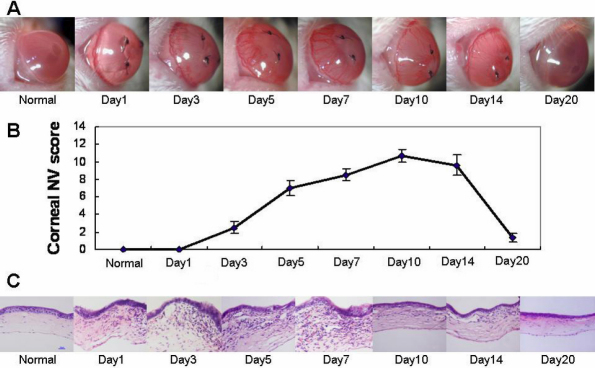
Suture-induced neovascularization and infiltration in murine corneas. The disease scores provided correspond to each time point.

**Figure 2:**
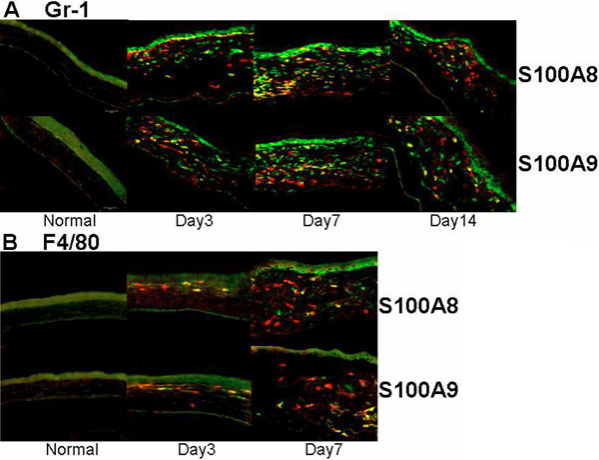
Immunostaining for S100A8, A9, and cellular markers in murine corneas. Neutrophils and macrophages were stained green via primary rabbit anti-Gr-1 (**A**) or anti-F4/80 (**B**) and FITC-conjugated secondary antibodies, while S100A8 or A9 were stained red via PE conjugated primary antibodies. Please note that the autofluorescence for corneal epithelium should not be misinterpreted as positive staining.

S100A proteins are reportedly produced mainly by macrophages and neutrophils, the two main cell types that infiltrated the corneas in CorNV models [3-5]. In our S-CorNV model, infiltration of these cells was detectable one day after suture placement and reached a maximum during days 3 and 7, after which it started to diminish, even when the suture was still present (Figure 1). The destruction of the corneal structure was mainly due to either the infiltration of inflammatory cells or neovascularization, or both. For example, the thickness of the cornea at day 10 returned to the normal range with the disappearance of previously infiltrated cells, even though substantial CorNV was still present. The growth and atrophy of the new vessels lagged behind the change of infiltration and retraction, suggesting that infiltrated cells might be the cause of CorNV. Immunohistochemistry analysis of the S-CorNV corneas showed that S100A8 and S100A9 were deposited in the neovascularized corneas (Figure 2). With both proteins, some of them co-located with neutrophils markers (i.e., Gr-1) while others with macrophage marker (i.e., F4/80), implying that these two proteins were produced in both neutrophils and macrophages. It should also be noted that not all neutrophils or macrophages stained positive for S100A8 or S100A9, demonstrating the heterogeneity of the infiltrated neutrophils or macrophages. This might also reflect the different activation status of these two classes of cells.

### Depletion of neutrophils abrogated S-CorNV and S100A8 and S100A9 production

**Figure 3:**
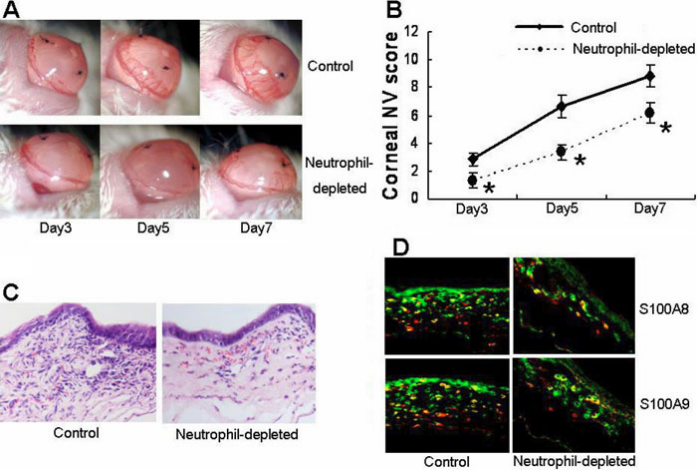
Effects of neutrophil depletion on various indexes associated with the pathogenesis of murine CorNV. **A** and **B** show the comparison of CorNV in normal and neutrophil-depleted mice at different times after suture placement (n=9 for **B**). **C** shows the infiltration under HE staining and **D** shows costaining of S100A8 or A9 with neutrophil marker Gr-1 at day 7. The examples shown are representative of nine (**A**, **B**) or three animals (**C**, **D**) in each group; the asterisk indicates a p<0.05 versus control.

**Figure 4:**
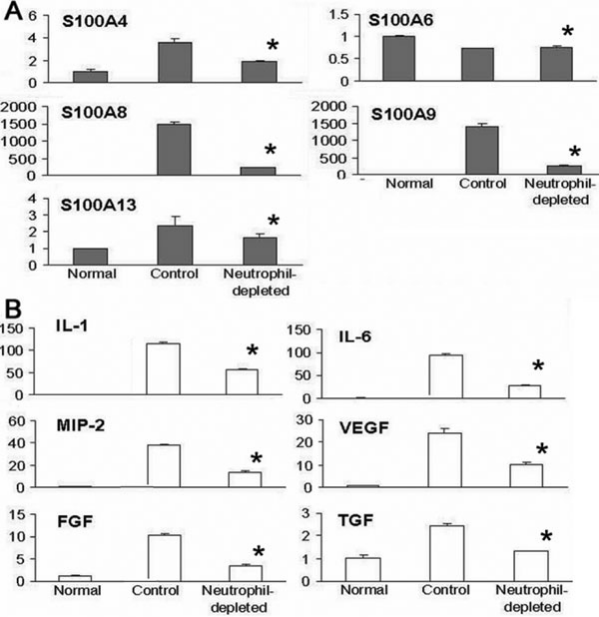
Effects of neutrophil depletion on the expression of S100A proteins and cytokines in neovascularized corneas at day 5 after suture placement. The relative level of each gene obtained by comparing with RPL5 in the normal group was set arbitrarily at 1.0 and the level in other groups was calculated accordingly. Five corneas were included in each group and the measures shown were for mean±standard deviation (n=5); the asterisk indicates a p<0.05 versus control.

In the CB-induced CorNV model, neutrophils were thought to be the main source of various inflammatory angiogenic factors [24], although macrophages are also a source of VEGF [25]. Gong et al. [24] reported that the depletion of neutrophils significantly inhibits corneal angiogenesis and that the inhibition was at least partially mediated by abrogating the VEGF and MIP pathways. We further showed that depletion of neutrophils decreased S100A8 and S100A9 expression in S-CorNV corneas compared to control S-CorNV corneas (Figure 3). The RT-QPCR analysis confirmed that neutrophil depletion in the mice significantly decreased, but did not completely abrogate the upregulation of the aforementioned genes (including *S100A8*, *S100A9*, *IL-1β*, *IL-6*, *MIP-2*, *FGF*, and *VEGFA*) in S-CorNV corneas (Figure 4). This implies that neutrophils are the main sources of S100A and other inflammatory molecules in S-CorNV.

### Depletion of S100A8 inhibited S-CorNV and inflammation

**Figure 5:**
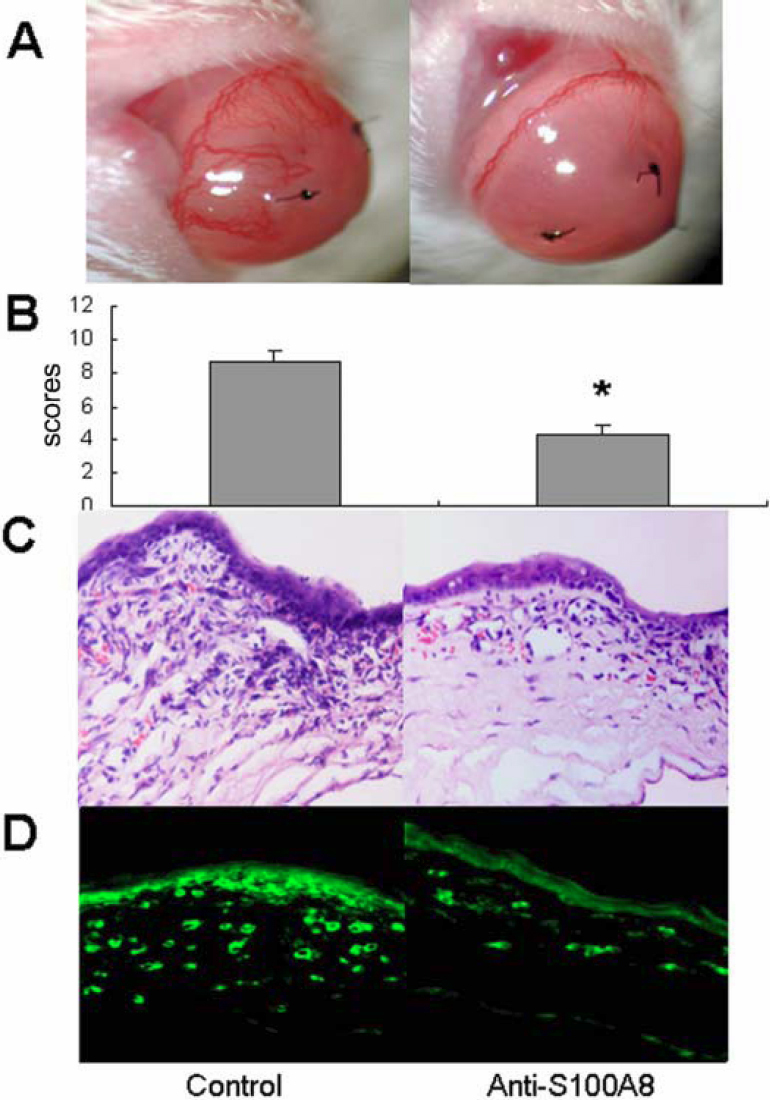
Effect of S100A8 neutralization on infiltration in neovascularized corneas at day 7 after suture placement. Shown are pictures taken under a slit lamp (**A**), disease scores (**B**), infiltration under HE staining (**C**), and staining for neutrophil marker Gr-1 (**D**); one representative cornea from three animals in each group; the asterisk indicates a p<0.05 versus control.

A further step was taken to check whether S100A8 was responsible for the neutrophil effect in the S-CorNV model. We found that subconjunctival application of a neutralizing antibody against S100A8 following suture placement significantly inhibited S-CorNV compared to the control group (Figure 5A,B). Surprisingly, S100A8 neutralization also decreased neutrophil infiltration (Figure 5C,D), implying that S100A8 might be at least partially responsible for neutrophil infiltration and CorNV development in the context of S-CorNV. Considering the above observation that S100A8 and S100A9 proteins are located in both neutrophils and macrophages (Figure 2), we suggest that S100A8 (and S100A9) produced by neutrophils attracted more neutrophils in turn, thus forming a positive feedback in certain stages of inflammatory CorNV. Thus, more in-depth studies about the relationship among macrophages, neutrophils, and these S100A proteins are necessary.

In summary, the above data shows that the changes of expression of the *S100A8* and *S100A9* genes, as well as several other cytokines, changed in concert with the growth and atrophy of new vessels in the S-CorNV model. These results suggest that *S100A8* and *S100A9* play a promoting role in the pathogenesis of inflammatory CorNV, just as *S100A4* and *S100A13* do in other neovascularization models [16,17,26,27]. More extensive studies are needed to define the suggested intrinsic relationship between *S100A8*, *S100A9*, and angiogenesis (or neovascularization). For example, it has been noted that S100A8, S100A9 and their heterodimer or tetramer manifested differential efficacy in terms of certain bioprocesses [28-30]. Thus comparative studies of mice that are deficient in *S100A8* or *S100A9* (e.g., gene knockout mice of such genes) will help to determine how these two genes take their effects in the development of CorNV under various conditions. This will also shed light on the physiologic significance of S100A8 and S100A9 proteins in corneas. A comprehensive in vitro and in vivo pro-angiogenic bioactivity assay of purified or recombinant *S100A8* or *S100A9* might help determine whether they are pro-angiogenic factors or are pro-angiogenic factor inducers. In fact, S100A8 and S100A9, either as is or in form of heterodimers [30-33], have been shown to promote death or permeability of vascular endothelial cells by binding to specific molecules on them [34,35]. Thus, the suggested promoting effect of S100A8 or S100A9 on S-CorNV must rely on other mechanisms that overcome the “negative” effect of these proteins on vascular endothelial cells. That said, the interactions of S100A8 or S100A9 with other angiogenic factors (e.g., IL-1, IL-6, VEGFA, FGF, and S100A4 or S100A13) need to be clarified.

To conclude, we found that *S100A8* and *S100A9* were involved in the inflammatory CorNV model. However, we do not know whether these genes work via pro-inflammatory or pro-angiogenic pathways under these conditions. Their net effect seems to be facilitating the growth of new vessels. Since both the anti-inflammation [18,36,37] and anti-angiogenic strategies [38,39] have been successful in controlling CorNV, we propose that these molecules might serve as novel targets for managing inflammatory CorNV. It is noteworthy that some S100A members have been reported to be involved in other pathological processes or diseases concerning the cornea, for example, S100A4 in keratonocus [40]. Recently, S100A6, S100A8, and S100A9 were found to be extensively expressed in pterygium tissue removed from patients [41,42]. We believe that with the expansion of our knowledge of S100A proteins, the list of involved S100A members and corneal diseases will grow. This will no doubt increase demand for investigation into the possible application of strategies targeting S100A genes in disease management.

## References

[1] Sellami D Abid S Bouaouaja G Ben Amor S Kammoun B Masmoudi M Dabbeche K Boumoud H Ben Zina Z Feki J Epidemiology and risk factors for corneal graft rejection. Transplant Proc 2007 39 2609 11 17954190 10.1016/j.transproceed.2007.08.020

[2] Chang JH Gabison EE Kato T Azar DT Corneal neovascularization. Curr Opin Ophthalmol 2001 12 242 9 11507336 10.1097/00055735-200108000-00002

[3] Sunderkötter C Beil W Roth J Sorg C Cellular events associated with inflammatory angiogenesis in the mouse cornea. Am J Pathol 1991 138 931 9 1707239 PMC1886108

[4] Sonoda KH Nakao S Nakamura T Oshima T Qiao H Hisatomi T Kinoshita S Ishibashi T Cellular events in the normal and inflamed cornea. Cornea 2005 24 S50 4 16227824 10.1097/01.ico.0000178736.35297.9d

[5] Gan L Fagerholm P Leukocytes in the early events of corneal neovascularization. Cornea 2001 20 96 9 11189012 10.1097/00003226-200101000-00018

[6] Hayashi K Hooper LC Detrick B Hooks JJ HSV immune complex (HSV-IgG: IC) and HSV-DNA elicit the production of angiogenic factor VEGF and MMP-9. Arch Virol 2009 154 219 26 19115032 10.1007/s00705-008-0303-7

[7] Biswas PS Banerjee K Kim B Kinchington PR Rouse BT Role of inflammatory cytokine-induced cyclooxygenase 2 in the ocular immunopathologic disease herpetic stromal keratitis. J Virol 2005 79 10589 600 16051851 10.1128/JVI.79.16.10589-10600.2005 PMC1182687

[8] Wallace GR John Curnow S Wloka K Salmon M Murray PI The role of chemokines and their receptors in ocular disease. Prog Retin Eye Res 2004 23 435 48 15219876 10.1016/j.preteyeres.2004.04.004

[9] Dana R Comparison of topical interleukin-1 vs tumor necrosis factor-alpha blockade with corticosteroid therapy on murine corneal inflammation, neovascularization, and transplant survival (an American Ophthalmological Society thesis). Trans Am Ophthalmol Soc 2007 105 330 43 18427620 PMC2258099

[10] Schäfer BW Heizmann CW The S100 family of EF-hand calcium-binding proteins: functions and pathology. Trends Biochem Sci 1996 21 134 40 8701470 10.1016/s0968-0004(96)80167-8

[11] Heizmann CW Fritz G Schafer BW S100 proteins: structure, functions and pathology. Front Biosci 2002 7 d1356 68 11991838 10.2741/A846

[12] Marenholz I Heizmann CW Fritz G S100 proteins in mouse and man: from evolution to function and pathology (including an update of the nomenclature). Biochem Biophys Res Commun 2004 322 1111 22 15336958 10.1016/j.bbrc.2004.07.096

[13] de Silva Rudland S Martin L Roshanlall C Winstanley J Leinster S Platt-Higgins A Carroll J West C Barraclough R Rudland P Association of S100A4 and osteopontin with specific prognostic factors and survival of patients with minimally invasive breast cancer. Clin Cancer Res 2006 12 1192 200 16489073 10.1158/1078-0432.CCR-05-1580

[14] Semov A Moreno MJ Onichtchenko A Abulrob A Ball M Ekiel I Pietrzynski G Stanimirovic D Alakhov V Metastasis-associated protein S100A4 induces angiogenesis through interaction with Annexin II and accelerated plasmin formation. J Biol Chem 2005 280 20833 41 15788416 10.1074/jbc.M412653200

[15] Ambartsumian N Klingelhofer J Grigorian M Christensen C Kriajevska M Tulchinsky E Georgiev G Berezin V Bock E Rygaard J Cao R Cao Y Lukanidin E The metastasis-associated Mts1(S100A4) protein could act as an angiogenic factor. Oncogene 2001 20 4685 95 11498791 10.1038/sj.onc.1204636

[16] Landriscina M Schinzari G Di Leonardo G Quirino M Cassano A D'Argento E Lauriola L Scerrati M Prudovsky I Barone C S100A13, a new marker of angiogenesis in human astrocytic gliomas. J Neurooncol 2006 80 251 9 16773219 10.1007/s11060-006-9189-y

[17] Hayrabedyan S Kyurkchiev S Kehayov I FGF-1 and S100A13 possibly contribute to angiogenesis in endometriosis. J Reprod Immunol 2005 67 87 101 16165218 10.1016/j.jri.2005.07.001

[18] Dana MR Zhu SN Yamada J Topical modulation of interleukin-1 activity in corneal neovascularization. Cornea 1998 17 403 9 9676913 10.1097/00003226-199807000-00011

[19] Shi L Reid LH Jones WD Shippy R Warrington JA Baker SC Collins PJ de Longueville F Kawasaki ES Lee KY Luo Y Sun YA Willey JC Setterquist RA Fischer GM Tong W Dragan YP Dix DJ Frueh FW Goodsaid FM Herman D Jensen RV Johnson CD Lobenhofer EK Puri RK Schrf U Thierry-Mieg J Wang C Wilson M Wolber PK Zhang L Amur S Bao W Barbacioru CC Lucas AB Bertholet V Boysen C Bromley B Brown D Brunner A Canales R Cao XM Cebula TA Chen JJ Cheng J Chu TM Chudin E Corson J Corton JC Croner LJ Davies C Davison TS Delenstarr G Deng X Dorris D Eklund AC Fan XH Fang H Fulmer-Smentek S Fuscoe JC Gallagher K Ge W Guo L Guo X Hager J Haje PK Han J Han T Harbottle HC Harris SC Hatchwell E Hauser CA Hester S Hong H Hurban! P, Jackson SA, Ji H, Knight CR, Kuo WP, LeClerc JE, Levy S, Li QZ, Liu C, Liu Y, Lombardi MJ, Ma Y, Magnuson SR, Maqsodi B, McDaniel T, Mei N, Myklebost O, Ning B, Novoradovskaya N, Orr MS, Osborn TW, Papallo A, Patterson TA, Perkins RG, Peters EH, Peterson R, Philips KL, Pine PS, Pusztai L, Qian F, Ren H, Rosen M, Rosenzweig BA, Samaha RR, Schena M, Schroth GP, Shchegrova S, Smith DD, Staedtler F, Su Z, Sun H, Szallasi Z, Tezak Z, Thierry-Mieg D, Thompson KL, Tikhonova I, Turpaz Y, Vallanat B, Van C, Walker SJ, Wang SJ, Wang Y, Wolfinger R, Wong A, Wu J, Xiao C, Xie Q, Xu J, Yang W, Zhang L, Zhong S, Zong Y, Slikker W Jr. The MicroArray Quality Control (MAQC) project shows inter- and intraplatform reproducibility of gene expression measurements. Nat Biotechnol 2006 24 1151 61 16964229 10.1038/nbt1239 PMC3272078

[20] Yang YH Dudoit S Luu P Lin DM Peng V Ngai J Speed TP Normalization for cDNA microarray data: a robust composite method addressing single and multiple slide systematic variation. Nucleic Acids Res 2002 30 e15 11842121 10.1093/nar/30.4.e15 PMC100354

[21] Tate MD Brooks AG Reading PC The role of neutrophils in the upper and lower respiratory tract during influenza virus infection of mice. Respir Res 2008 9 57 18671884 10.1186/1465-9921-9-57 PMC2526083

[22] Dallas PB Gottardo NG Firth MJ Beesley AH Hoffmann K Terry PA Freitas JR Boag JM Cummings AJ Kees UR Gene expression levels assessed by oligonucleotide microarray analysis and quantitative real-time RT-PCR–how well do they correlate? BMC Genomics 2005 6 59 15854232 10.1186/1471-2164-6-59 PMC1142514

[23] Yuen T Wurmbach E Pfeffer RL Ebersole BJ Sealfon SC Accuracy and calibration of commercial oligonucleotide and custom cDNA microarrays. Nucleic Acids Res 2002 30 e48 12000853 10.1093/nar/30.10.e48 PMC115302

[24] Gong Y Koh DR Neutrophils promote inflammatory angiogenesis via release of preformed VEGF in an in vivo corneal model. Cell Tissue Res 2010 339 437 48 20012648 10.1007/s00441-009-0908-5

[25] Lu P Li L Wu Y Mukaida N Zhang X Essential contribution of CCL3 to alkali-induced corneal neovascularization by regulating vascular endothelial growth factor production by macrophages. Mol Vis 2008 14 1614 22 18776949 PMC2529469

[26] Massi D Landriscina M Piscazzi A Cosci E Kirov A Paglierani M Di Serio C Mourmouras V Fumagalli S Biagioli M Prudovsky I Miracco C Santucci M Marchionni N Tarantini F S100A13 is a new angiogenic marker in human melanoma. Mod Pathol 2010 23 804 13 20208480 10.1038/modpathol.2010.54 PMC2882157

[27] Boye K Maelandsmo GM S100A4 and metastasis: a small actor playing many roles. Am J Pathol 2010 176 528 35 20019188 10.2353/ajpath.2010.090526 PMC2808059

[28] Vogl T Tenbrock K Ludwig S Leukert N Ehrhardt C van Zoelen MA Nacken W Foell D van der Poll T Sorg C Roth J Mrp8 and Mrp14 are endogenous activators of Toll-like receptor 4, promoting lethal, endotoxin-induced shock. Nat Med 2007 13 1042 9 17767165 10.1038/nm1638

[29] Leukert N Vogl T Strupat K Reichelt R Sorg C Roth J Calcium-dependent tetramer formation of S100A8 and S100A9 is essential for biological activity. J Mol Biol 2006 359 961 72 16690079 10.1016/j.jmb.2006.04.009

[30] Eue I Konig S Pior J Sorg C S100A8, S100A9 and the S100A8/A9 heterodimer complex specifically bind to human endothelial cells: identification and characterization of ligands for the myeloid-related proteins S100A9 and S100A8/A9 on human dermal microvascular endothelial cell line-1 cells. Int Immunol 2002 14 287 97 11867565 10.1093/intimm/14.3.287

[31] Hunter MJ Chazin WJ High Level Expression and Dimer Characterization of the S100 EF-hand Proteins, Migration Inhibitory Factor-related Proteins 8 and 14. J Biol Chem 1998 273 12427 35 9575199 10.1074/jbc.273.20.12427

[32] Ehlermann P Eggers K Bierhaus A Most P Weichenhan D Greten J Nawroth PP Katus HA Remppis A Increased proinflammatory endothelial response to S100A8/A9 after preactivation through advanced glycation end products. Cardiovasc Diabetol 2006 5 6 16573830 10.1186/1475-2840-5-6 PMC1475836

[33] Robinson MJ Tessier P Poulsom R Hogg N The S100 family heterodimer, MRP-8/14, binds with high affinity to heparin and heparan sulfate glycosaminoglycans on endothelial cells. J Biol Chem 2002 277 3658 65 11723110 10.1074/jbc.M102950200

[34] Viemann D Strey A Janning A Jurk K Klimmek K Vogl T Hirono K Ichida F Foell D Kehrel B Gerke V Sorg C Roth J Myeloid-related proteins 8 and 14 induce a specific inflammatory response in human microvascular endothelial cells. Blood 2005 105 2955 62 15598812 10.1182/blood-2004-07-2520

[35] Viemann D Barczyk K Vogl T Fischer U Sunderkotter C Schulze-Osthoff K Roth J MRP8/MRP14 impairs endothelial integrity and induces a caspase-dependent and -independent cell death program. Blood 2007 109 2453 60 17095618 10.1182/blood-2006-08-040444

[36] Jin Y Arita M Zhang Q Saban DR Chauhan SK Chiang N Serhan CN Dana R Anti-angiogenesis effect of the novel anti-inflammatory and pro-resolving lipid mediators. Invest Ophthalmol Vis Sci 2009 50 4743 52 19407006 10.1167/iovs.08-2462 PMC2763387

[37] Cursiefen C Maruyama K Jackson DG Streilein JW Kruse FE Time course of angiogenesis and lymphangiogenesis after brief corneal inflammation. Cornea 2006 25 443 7 16670483 10.1097/01.ico.0000183485.85636.ff

[38] Shakiba Y Mansouri K Arshadi D Rezaei N Corneal neovascularization: molecular events and therapeutic options. Recent Pat Inflamm Allergy Drug Discov 2009 3 221 31 19702562 10.2174/187221309789257450

[39] Qazi Y Maddula S Ambati BK Mediators of ocular angiogenesis. J Genet 2009 88 495 515 20090210 10.1007/s12041-009-0068-0 PMC3306772

[40] Nielsen K Vorum H Fagerholm P Birkenkamp-Demtroder K Honore B Ehlers N Orntoft TF Proteome profiling of corneal epithelium and identification of marker proteins for keratoconus, a pilot study. Exp Eye Res 2006 82 201 9 16083875 10.1016/j.exer.2005.06.009

[41] Riau AK Wong TT Beuerman RW Tong L Calcium-binding S100 protein expression in pterygium. Mol Vis 2009 15 335 42 19223989 PMC2642841

[42] Jaworski CJ Aryankalayil-John M Campos MM Fariss RN Rowsey J Agarwalla N Reid TW Dushku N Cox CA Carper D Wistow G Expression analysis of human pterygium shows a predominance of conjunctival and limbal markers and genes associated with cell migration. Mol Vis 2009 15 2421 34 19956562 PMC2785720

